# Selection of established tumour cells through narrow diameter micropores enriches for elevated Ras/Raf/MEK/ERK MAPK signalling and enhanced tumour growth

**DOI:** 10.1080/21541248.2020.1780108

**Published:** 2020-06-22

**Authors:** Dominika a Rudzka, Susan Mason, Matthew Neilson, Lynn McGarry, Gabriela Kalna, Ann Hedley, Karen Blyth, Michael F. Olson

**Affiliations:** a Cancer Research UK Beatson Institute, Glasgow, UK; b Institute of Cancer Sciences, University of Glasgow, Glasgow, UK; c Department of Chemistry and Biology, Ryerson University, Toronto, ON, Canada

**Keywords:** Migration, cytoskeleton, gene expression, RAS, tumor, metastasis

## Abstract

As normal cells become cancer cells, and progress towards malignancy, they become progressively softer. Advantages of this change are that tumour cells become more deformable, and better able to move through narrow constraints. We designed a positive selection strategy that enriched for cells which could move through narrow diameter micropores to identify cell phenotypes that enabled constrained migration. Using human MDA MB 231 breast cancer and MDA MB 435 melanoma cancer cells, we found that micropore selection favoured cells with relatively higher Ras/Raf/MEK/ERK mitogen-activated protein kinase (MAPK) signalling, which affected actin cytoskeleton organization, focal adhesion density and cell elasticity. In this follow-up study, we provide further evidence that selection through micropores enriched for cells with altered cell morphology and adhesion. Additional analysis of RNA sequencing data revealed a set of transcripts associated with small cell size that was independent of constrained migration. Gene set enrichment analysis identified the ‘matrisome’ as the most significantly altered gene set linked with small size. When grown as orthotopic xenograft tumours in immunocompromised mice, micropore selected cells grew significantly faster than Parent or Flow-Sorted cells. Using mathematical modelling, we determined that there is an interaction between 1) the cell to gap size ratio; 2) the bending rigidity of the cell, which enable movement through narrow gaps. These results extend our previous conclusion that Ras/Raf/MEK/ERK MAPK signalling has a significant role in regulating cell biomechanics by showing that the selective pressure of movement through narrow gaps also enriches for increased tumour growth *in vivo*.

## Introduction

Metastasis is the leading factor contributing to patient deaths from cancer, said to play a role in as many as 90% of the total [1,2]. In fact, some forms of the disease, including breast cancer and melanoma, are not particularly threatening at their site of origin. Instead, these cancers cause devasting harm after having spread to distant sites and establishing secondary tumours, particularly when the metastatic tumours are located at essential tissues/organs such as liver, lung, brain or bones [3,4].

The metastatic process consists of several stages; initially individual cells or clusters detach from a primary tumour and begin invading local tissues [5]. The invasive tumour cells pass through robust physical barriers, including dense tumour-associated extracellular matrix (ECM), basement membranes and endothelial cell layers as they intravasate into the blood and lymphatic vessels that carry them around the body. Some of the tumour cells that have survived the journey will extravasate and establish micrometastatic colonies that may grow into macrometastases, and eventually into full secondary tumours. At each step of the metastatic process, positive selective pressures favour those cells with advantageous phenotypes that enable survival and proliferation under adverse conditions.

During the early stages of local invasion and intravasation, tumour cells will likely be confronted with narrow physical constraints that must be traversed to move forward. Gaps in three-dimensional (3D) ECM can take the form of small diameter pores or longer tunnels, which may often be narrower than the invading cells [6,7]. These micropores and tracks pre-exist in various healthy tissues, such as within brain perivascular spaces [8], along blood vessels [9], and between muscle and nerve fibres [7]. Although there may also be pre-existing gaps or tunnel-like tracks within cross-linked collagen fibres in the ECM [10], cells may encounter physical constraints imposed by narrow diameter pores, which can be relieved by the tumour cells themselves, or by tumour-associated stromal cells that have been shown to be significant participants in tumour cell spread [11,12]. The narrowness of the space or track through which a cell may be passing can be increased by enzymatic ECM degradation to enable tumour cell invasion, for example [13]. In addition, the application of physical force produced by invading cells or co-operating cancer-associated cells can increase the size of spaces within the physically constraining environment to enable passage [14].

At the same time that the ECM may be modified to allow tumour cells to move through small gaps, the same physical constraints may be a selective pressure that enriches for cells with phenotypes that enable movement through narrow spaces. One such property is cell elasticity; changes in the pliability of a cell that enable dynamic shape changes would also allow it to squeeze through physical constraints, which can play a significant role in local tissue invasion and eventual metastasis [15]. Consistent with the concept that cellular mechanical properties play a significant role in cancer, tumour cells have been shown to be softer than normal cells, and highly metastatic cells are even softer than less metastatic cancer cells [16–19]. These changes in physical properties contribute to the early stages of the metastatic process by increasing deformability to help cells pass through physical constraints [16,20].

Established tumour cell lines are critically important models that facilitate studies aiming to identify how cancer cells differ from normal cells. Typically, tumour samples are used as the source material to grow out individual clones from single originating cells, which means that all the cells in a given established line initially are genetically identical. Analytical approaches that examine properties at the single cell level, such as high content image analysis, mapping of chromatin organization and transcriptomics [21–23], have revealed the extensive phenotypic diversity in many properties and behaviours of established cell lines. By imposing a selective pressure, phenotypes that confer an advantage under defined conditions may be enriched, similar to the way that tumour cell heterogeneity enables adaptions to the changing environmental conditions that occur during cancer progression [24,25].

In our publication by Rudzka *et al* [26]., we used microporous membranes to select for cells that were relatively more efficient at moving through narrow diameter (3 µm) gaps from the established MDA MB 231 human breast cancer and MDA MB 435 melanoma cell lines. Following three consecutive rounds of selection, three independent populations of Pore-Selected (Sel) were established. In addition, cell sorting by flow cytometry was used to enrich for small sized MDA MB 231 cells, and three independent populations of Flow-Sorted (FS) cells were isolated. These Sel and FS populations were then compared to the starting Parent populations to identify phenotypes that were specifically associated with the ability of cells to move through narrow pores, independent of properties linked to small cell size. Then, by comparing the differences between Parent and Sel MDA MB 231 breast cancer cells with differences between Parent and Sel MDA MB 435 melanoma cells, we were able to identify phenotypes associated with pore-selection that were shared between diverse cancer cell types. In this way, we determined that small cell and nucleus size were not sufficient to enable movement through small gaps. Although Pore-Selected cells did have smaller nuclei than Parent cells, Flow-Sorted cells had equally small nuclei. Independent of the means of isolation, the small nuclei of both Pore-Selected and Flow-Sorted cells contained less DNA, had fewer chromosomes and were comparably stiffer than Parent cell nuclei. Instead, significant changes in the actin-myosin cytoskeleton, including reduced levels of filamentous actin (F-actin) and decreased F-actin anisotropy, which led to decreased focal adhesion density, increased migration velocity and cell stiffness were key properties of Pore-Selected cells. To identify the molecular basis for the differences in ability to move through small pores, we performed RNA sequencing to identify differences in transcript expression [27]. Comparison of the transcripts that were associated with the ability of cells to move through narrow pores, and not with small cell size, revealed parallels with gene sets linked to changes in Ras/Raf/MEK/ERK Mitogen-activated protein kinase (MAPK) signalling. Inhibition of MAPK/ERK kinase (MEK) activity with selective small molecule inhibitors in Pore-Selected cells led to a reversal in the actin cytoskeleton effects, focal adhesion density, migration velocity and cell stiffness. Conversely, by increasing Ras/Raf/MEK/ERK MAPK signalling in unselected Parental cells via ectopic expression of activated KRas in MDA MB 231 cells or oncogenic BRaf in MDA MB 435 cells, there were similar changes in the actin cytoskeleton, focal adhesions and cell elasticity as were seen in Pore-Selected cells. These findings revealed that Ras/Raf/MEK/ERK MAPK signalling has a central role in the regulation of cell elasticity through its effects on the actin cytoskeleton.

In this present study, we extend these previous observations to show that there were morphological and adhesion differences which were more pronounced in Pore-Selected Sel cells than in Flow-Sorted FS cells, relative to the starting Parent cell populations. Analysis of RNA sequencing results revealed that in addition to a gene set we previously found to be linked with the ability of Pore-Selected cells to move through narrow diameter gaps, we also identified a 547 gene set associated with small cell size. The two most significant classes of transcripts in the small cell gene set were linked with the extracellular matrix and the nucleosome. When the three populations of MDA MB 231 cells were grown as orthotopic xenograft tumours, Pore-Selected Sel tumours grew significantly faster than Parent or FS tumours, indicating that the properties which enable more efficient movement through narrow pores also contribute to tumour growth *in vivo*. Finally, by mathematical modelling the two independent variables of cell stiffness and cell-to-gap ratio, we determined that these two factors co-operate to facilitate migration through narrow pores. These results reveal that the selective pressure that enriched for cells able to move through narrow constrictions led to the selection of small and pliable cells, which also have the property of enhanced tumour growth *in vivo*.

## Results

### Cell selection by passage through narrow diameter pores or cell sorting

**Figure 1.**
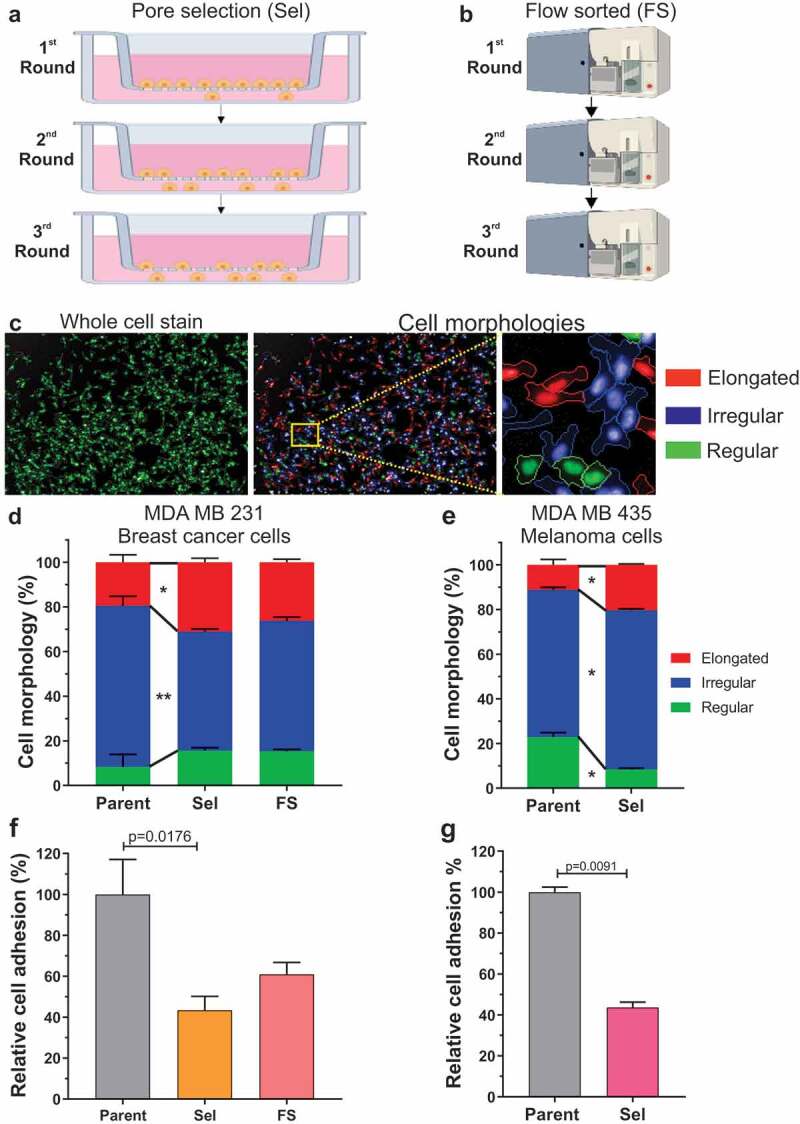
Small diameter pore-selection enriches for invasive and metastatic cell behaviours. (a). Schematic diagram of pore-selection strategy. Cells (106) were placed in serum-free medium in 7.5 cm diameter tissue culture inserts with microporous membranes (3 µm pore diameter), and allowed to migrate for 5 days towards serum-containing medium below. Successful cells were collected, expanded and re-plated twice more as above. Three separate Pore-Selected (Sel) populations were isolated for each cell line. Created with Biorender. (b). Schematic diagram of cell sorting by flow cytometry. Independent small diameter MDA MB 231 Flow-Sorted (FS) populations were obtained by gating with low forward scatter and side scatter parameters using a FACSAria Fusion sorter. FS cells were grown using standard tissue culture conditions to expand the isolated sorted cell populations, followed by two additional rounds of sorting as described above. Created with Biorender. (c). An Operetta High-Content Imaging System was used to determine cell morphological features. Cells were seeded in black 96-well plates at 2 × 10^4^ cells per well and grown overnight, then fixed with 4% para-formaldehyde, permeabilized with 0.5% Triton X-100 and stained with 0.15 µg/ml DAPI and Cellomics® Whole Cell Stain Plates were imaged on an Operetta High-Content Imaging System and data were analysed using a Columbus™ Image Data Storage and Analysis System (PerkinElmer). Multiparametric analysis was used to classify cells into three morphology categories: elongated (red), irregular (blue) and regular (green). (d). Percentages of each cell morphology class for Parent (n = 3), Sel (n = 9) and FS (n = 9) MDA MB 231 populations. Kruskal-Wallis test with Dunn’s multiple comparisons (* = p < 0.05, ** = p < 0.01). Means ± SEM. (e). Percentages of each cell morphology class for Parent (n = 3) and Sel (n = 6) MDA MB 435 populations. Mann-Whitney U test of significance (* = p < 0.05). Means ± SEM. (f). Relative cell adhesion determined by counting cell numbers remaining after 5 minutes of Dispase treatment, normalized to the mean determined for Parent MDA MB 231 cells (n = 4). The number of experimental replicates were Sel (n = 12) and FS (n = 12). Kruskal-Wallis test with Dunn’s multiple comparisons (* = p < 0.05). Means ± SEM. (g). Relative cell adhesion normalized to the mean determined for Parent MDA MB 435 cells (n = 3). The number of experimental replicates were Sel (n = 9). Mann-Whitney U test of significance (** = p < 0.01). Means ± SEM

In order to identify properties that enable cancer cells to move through narrow physical constraints, similar to what they might encounter while passing through dense extracellular matrix, we previously used tissue culture inserts with microporous membranes (3 µm average diameter pores) to isolate MDA MB 231 D3H2LN luciferase breast cancer (abbreviated MDA MB 231; reference [28]) or MDA MB 435 melanoma cells capable of overcoming the physical restrictions to their passage (Figure 1(a)) [26]. Both cell lines have activating mutations in the Ras/Raf/MEK/ERK MAPK signalling pathway; MDA MB 231 cells have oncogenic KRAS^G13D^ and weakly activating BRAF^G464^ ^V^ mutations while MDA MB 435 cells have strongly activated BRAF^V600E^. Cells were plated in serum-free medium, and those cells that moved through microporous membranes to the serum-containing medium below were collected, expanded, and then subjected to two additional rounds of selection. In total, three independent populations of Pore-Selected (abbreviated Sel) cells from each established tumour cell line were isolated after three rounds of enrichment. We observed that the Pore-Selected populations were smaller on average than the Parent cells from which that had been selected [26], so for comparison purposes, three consecutive rounds of cell sorting by flow cytometry (Flow-Sorted; FS) were used to enrich for three independent populations of small diameter MDA MB 231 cells (Figure 1(b)). We determined that Pore-Selected cells were significantly more efficient at passage through narrow diameter micropores than either Parent or FS populations, which was associated with changes in the organization of the actin-myosin cytoskeleton that resulted in decreased cell stiffness [26].

Single cell high content imaging was used to classify cells into three morphology categories; first fluorescent whole cell staining was used for visualization and quantification of morphological parameters (Figure 1(c), left), which were then used to cluster cells into elongated (red), irregular (blue) or regular (green) groups (Figure 1(c), centre and right). Each of the Parent, 3 Sel populations and 3 FS MDA MB 231 populations were assayed three times, with the mean results of the three Sel and FS populations being combined in a single category in Figure 1(d). Pore-selection resulted in a significant decrease in the proportion of irregular cells and concomitant increase in elongated cells (Figure 1(d)). Flow sorting resulted in similar trends, but the proportions of cells in each category were not significantly different from Parent or Sel populations. These results suggest that the major influence on cell morphology is the enrichment for small cell size, plus an additional contribution from the cytoskeleton differences in the Sel populations that resulted in changes in cell elasticity [26]. Similar to the observations with the MDA MB 231 cells, pore-selection of MDA MB 435 melanoma cells significantly increased the proportion of elongated cells, while decreasing the proportions of irregular and regular morphologies (Figure 1(e)).

The organization of the filamentous actin (F-actin) cytoskeleton is a major factor regulating cancer cell migration, and in determining cell morphology and adhesion properties [29]. We found that the Pore-Selected MDA MB 231 cells had lower focal adhesion densities (*i.e*. focal adhesion number per unit area) than either Parent or FS populations, an observation that was paralleled by lower focal adhesion densities in MDA MB 435 Sel populations relative to Parent cells [26]. We sought to determine whether the reduced focal adhesion densities resulted in weaker substrate adhesion using an assay based upon the relative number of cells remaining following a brief exposure to a dilute collagenase solution. Each of the Parent, 3 Sel populations and 3 FS MDA MB 231 populations were assayed four times, with the Sel and FS results being pooled in Figure 1(f). The Sel populations were significantly more sensitive to treatment with collagenase relative to Parent cells (Figure 1(f)), consistent with their reduced focal adhesion density. The relative numbers of Flow-Sorted cells were also reduced, but not significantly, suggesting that the major influence on adhesion is cell size, with an additional contribution from the lower focal adhesion density in the Sel populations compared to either Parent or FS cells [26]. Similar to the observations with the MDA MB 231 cells, pore-selection of MDA MB 435 melanoma cells significantly decreased cell adhesion (Figure 1(g)).

### Pore-selected cells have elevated Ras/Raf/MEK/ERK MAPK signalling as revealed by RNA sequencing

**Figure 2.**
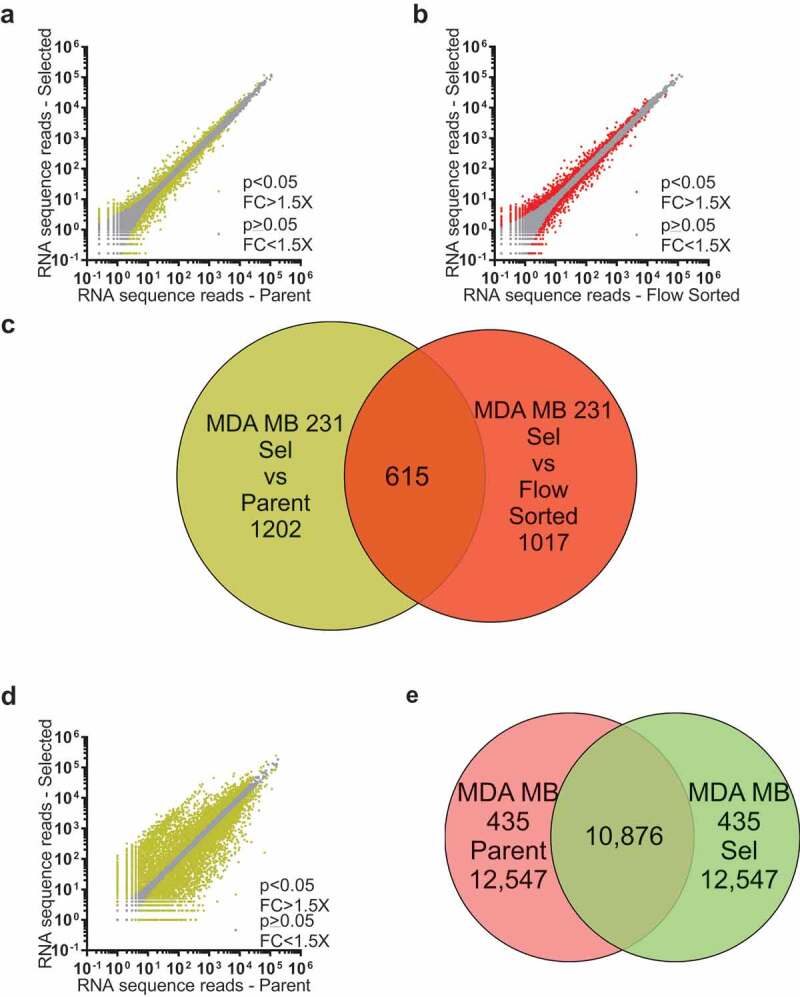
RNA sequencing comparisons of MDA MB 231 Parent, Selected and Flow-Sorted cells, and MDA MB 435 Parent and Selected cells. (a). Log10-log10 scatter plots of mean RNAseq reads as described in Rudzka *et al* [27]. for Parent vs Selected MDA MB 231 cells, where p < 0.05 and fold-change (FC) differences > 1.5X indicated with yellow dots, p ≥ 0.05 or FC ≤ 1.5X indicated with grey dots. (b). Log10-log10 scatter plots of mean RNAseq reads for Flow-Sorted vs Selected MDA MB 231 cells, where p < 0.05 and FC > 1.5X indicated with red dots, p ≥ 0.05 or FC ≤ 1.5X indicated with grey dots. (c). Venn diagram indicating the 615 gene overlap (Table S3) between the 1817 genes that were different by p < 0.05 and FC > 1.5X between Parent and Selected MDA MB 231 cells (yellow circle; Table S1), and the 1632 genes that were different by p < 0.05 and FC > 1.5X between flow-sorted and selected MDA MB 231 cells (red circle; Table S2). (d). Log10-log10 scatter plots of mean RNAseq reads for Parent vs Selected MDA MB 435 cells, where p < 0.05 and FC > 1.5X indicated with yellow dots, p ≥ 0.05 or FC ≤ 1.5X indicated with grey dots. (e). Venn diagram indicating the 10,876 gene overlap (Table S4) that were different by p < 0.05 and FC > 1.5X between parent (red circle) and selected (green circle) MDA MB 435 cells

**Figure 3.**
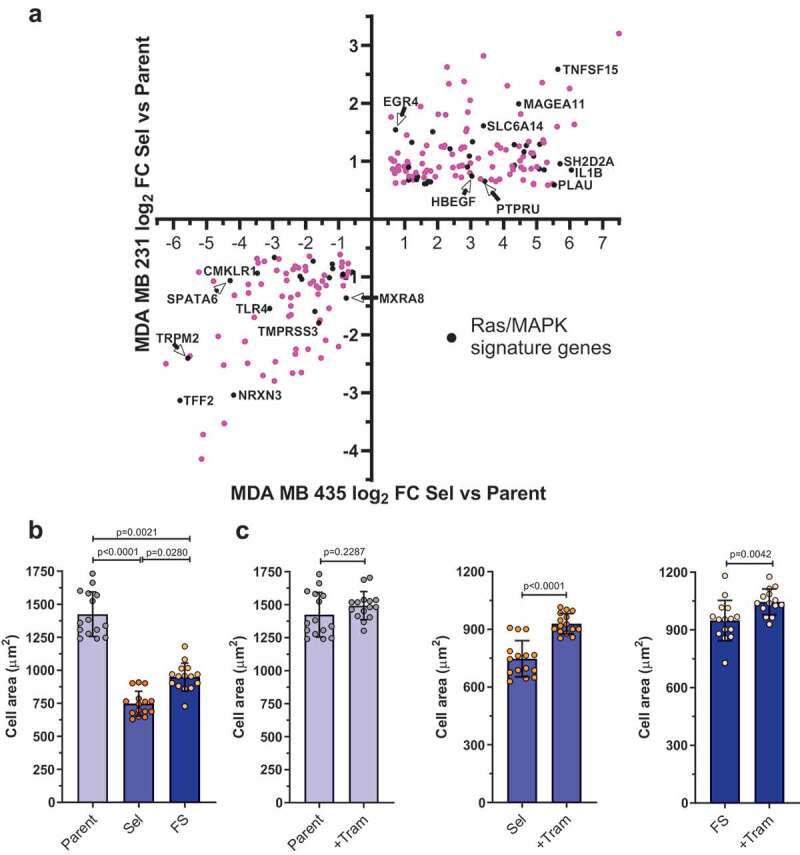
Scatter plot of pore-invasion gene set expression differences between parent and pore-selected MDA MB 231 breast cancer cells versus parent and pore-selected MDA MB 435 melanoma cells. To visualize the distribution of the Ras/Raf/MEK/ERK MAPK regulated transcripts (black symbols) within the 214 invasion gene set (pink symbols; Table S5), the log2 fold change differences in expression between parent and pore-selected MDA MB 231 cells were plotted versus parent and pore-selected MDA MB 435 cells. (b). Graph depicts mean (± SEM) cell area for parental, selected and flow-sorted MDA MB 231 cells treated with DMSO. Statistical significance determined by Kruskal – Wallis followed by Dunn’s multiple comparison test. (c). Graphs depict mean (± SEM) cell area for Parental (left), Selected (centre) and Flow-Sorted (right) MDA MB 231 cells treated with DMSO or Trametinib (+Tram) as indicated. Each dot represents an average from n = 6 fields per well, from at least 200 cells per field. Data pooled from n = 3 independent experiments. Statistical significance determined by Mann – Whitney test

We reasoned that the properties of Pore-Selected and Flow-Sorted cells that differentiated them from Parent MDA MB 231 or MDA MB 435 cells were likely due to transcriptional differences between the populations, one reason being that they were stably maintained over time and multiple cell passages [26]. RNA sequencing (RNAseq) was performed on polyA+ enriched RNA from 4 replicate dishes of Parent MDA MB 231 cells, two Pore-Selected isolates (Sel2, Sel3; three replicates each) and two Flow-Sorted isolates (FS1, FS2; three replicates each), as described in Rudzka *et al* [27]. To visualize how gene expression differed, the mean number of sequence reads for each transcript from the Parent replicates and the combined Sel MDA MB 231 cell replicates was graphed, with differences between the populations that were > ±1.5 fold and with statistical significance <0.05 (Figure 2(a)) indicated using yellow symbols for the 1817 transcripts that met both criteria. Similarly, the mean number of sequence reads for each transcript from Flow-Sorted vs Sel cells was graphed, with differences between the populations that were > ±1.5 fold and with statistical significance <0.05 (Figure 2(b)) indicated using red symbols for the 1632 transcripts that met both criteria. To identify a set of genes that could be associated with properties of cells that efficiently moved through narrow diameter pores (*e.g*. F-actin changes, reduced focal adhesion density, increased migration speed, lower cell stiffness, *etc*.), independent of changes in gene expression associated with small cell size, the 1817 significantly altered transcripts from the Parent vs Sel (Figure 2(a), Table S1) comparison were matched to the 1632 significantly altered transcripts from the Flow-Sorted vs Sel (Figure 2(b), Table S2) comparison, revealing 615 transcripts in common (Figure 2(c), Table S3). To further validate the MDA MB 231 invasive gene set, a similar comparison was made between 4 replicate dishes of Parent MDA MB 435 cells and two Pore-Selected isolates (Sel1, Sel2; 4 replicates each). Using the same criteria of > ±1.5 fold-change and p < 0.05 statistical cut-off, the number and magnitude of gene expression differences between MDA MB 435 Parent and Pore-Selected Sel1 and Sel2 populations were greater, with 10,876 differentially expressed genes from the 23,423 identified transcripts, representing 46% of the total (Figure 2(d,e); Table S4). When compared with the 615 ‘invasive’ gene signature (Figure 2(c), Table S3), there were 214 genes that were consistently associated with constrained migration ability, which have been defined as the pore-invasion gene set (**Table S5**) [26]. When the 615 transcripts from the MDA MB 231 comparisons in **Table S3** was compared with the 189 Gene Set Enrichment Analysis [30] (GSEA) defined ‘Oncogenic Signature’ gene sets, 5 of the 6 most statistically significant oncogenic signature gene sets were associated with KRAS signalling (**Table S6**). Similarly, when the consensus 214 pore-invasion gene set from **Table S5** was compared with the GSEA 189 ‘Oncogenic Signature’ gene sets, 4 of the 5 most statistically significant gene sets were associated with KRAS signalling (**Table S7**). In total, 49 genes from the 214 pore-invasion gene set were statistically significantly associated with KRAS, BRAF, and/or Mitogen Activated Protein Kinase (MAPK)-linked oncogenic signature gene sets (**Table S8**) [26], 18 expressed at lower levels and 31 at higher levels in Sel2 and Sel2 populations relative to Parent MDA MB 231 cells, and in Sel1 and Sel2 isolates relative to Parent MDA MB 435 cells. By plotting the log_2_ fold-change (FC) of each gene for the MDA MB 435 Parent vs Sel comparison on the X-axis and for the MDA MB 231 Parent vs Sel comparison on the Y-axis, it can be seen that the up and down-regulation in expression of the Ras/Raf/MEK/ERK regulated genes is consistent (Figure 3(a), black symbols). In agreement with elevated Ras/Raf/MEK/ERK signalling contributing to the significantly smaller area of Sel cells relative to Parent MDA MB 231 cells (Figure 3(b)), treatment with the MEK inhibitor Trametinib (Tram) had no significant effect on Parent cell areas but significantly increased Sel cell areas by 24% (Figure 3(c)). There was a smaller 10% increase in the FS cell area following Trametinib treatment.

### Gene expression changes associated with small cell size identified by RNA sequencing

**Figure 4.**
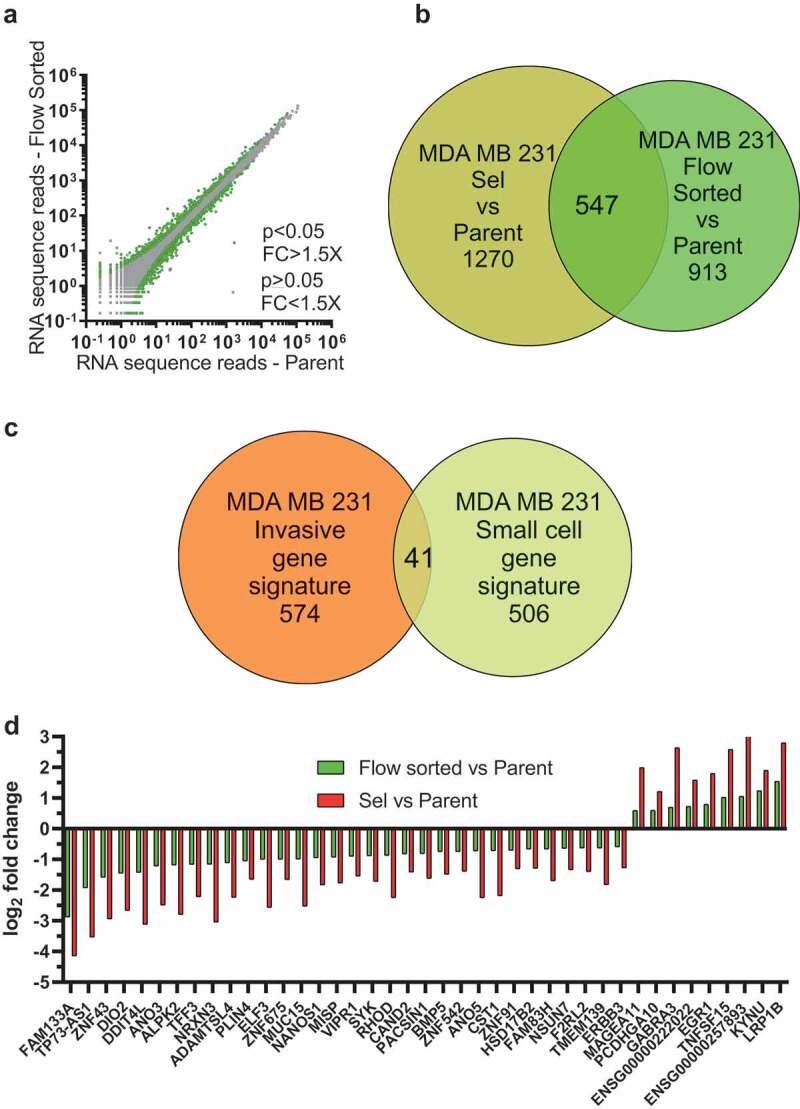
Identification of a small cell size gene set. (a). Log10-log10 scatter plots of mean RNAseq reads for Flow-Sorted vs Parent MDA MB 231 cells, where p < 0.05 and FC > 1.5X indicated with green dots, p ≥ 0.05 or FC ≤ 1.5X indicated with grey dots. (b). Venn diagram indicating the 547 gene overlap (Table S10) between the 1817 genes that were different by p < 0.05 and FC > 1.5X between Parent and Selected cells (olive green circle; Table S1), and the 1460 genes that were different by p < 0.05 and FC > 1.5X between Flow-Sorted and Parent MDA MB 231 cells (light green circle; Table S9). (c). Venn diagram indicating the 41 gene overlap (Table S11) between the 615 invasive gene signature set (Table S3) and the 547 small cell size gene signature set (Table S10). (d). To visualize the fold-change differences in gene expression for the 41 common transcripts identified in the pore-invasion and small cell size gene signature sets (Table S11), log2 fold change differences between Parent and Flow-Sorted MDA MB 231 cells (green bars) and between Parent and Pore-Selected MDA MB 231 cells (red bars) were plotted

The patterns of differential gene expression determined from Pore-Selected Sel versus Parent cells (Figure 2(a)) compared to Sel vs Flow-Sorted cells (Figure 2(b)) revealed a gene set associated with movement through narrow diameter pores (Figure 2(c)) that was independent of small cell size. This raised the possibility that there would be a separate set of genes differentially expressed in small cells, independent of their mode of selection (*i.e*. pore-selection or flow sorting). To visualize how gene expression differed between Parent and Flow-Sorted FS MDA MB 231 cells, the mean number of sequence reads for each transcript was graphed, with differences between the populations that were > ±1.5 fold and with statistical significance <0.05 indicated using green symbols for the 1460 transcripts that met both criteria (Figure 4(a), Table S9). The 1817 significantly altered transcripts from the Parent vs Sel (Figure 2(a), Table S1) comparison were matched to the 1460 significantly altered transcripts from the Parent vs Flow-Sorted (Figure 4(a), Table S9) comparison, revealing 547 transcripts in common (Figure 4(b), Table S10) associated with small cell size. To validate that the 615 invasive gene set (Figure 2(c), Table S3) represented transcripts associated with the properties of cells selected through narrow diameter pores (*e.g*. F-actin changes, reduced focal adhesion density, increased migration speed, lower cell stiffness, *etc*.), and which were independent of changes in gene expression associated with small cell size, they were compared to the 547 small cell gene set (Figure 4(b), Table S10), revealing 41 common genes (**Table S11**). When the fold changes in gene expression were compared, the significant changes observed for Flow-Sorted vs Parent were even larger for Sel vs Parent (Figure 4(d)), such that the changes in gene expression between Flow-Sorted and Sel were also significant and > ±1.5 fold (Figure 2(b)). We concluded that these 41 genes were associated with both the invasive and small gene sets, but could be differentiated between the gene sets based on the magnitude of the differences in their expression in Flow-Sorted (smaller changes) or Pore-Selected Sel (larger changes) cells relative to Parent cells.

**Figure 5.**
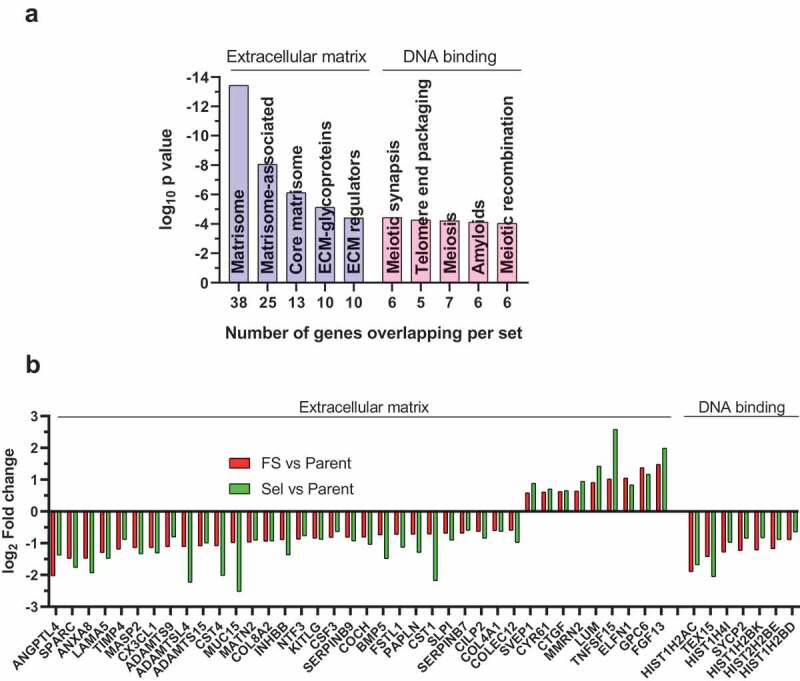
Gene set enrichment analysis to identify canonical pathways associated with the small cell size gene set. (a). Most significant GSEA ‘canonical pathways’ identified for the MDA MB 231 small sell size gene set (Table S12). (b). To visualize the fold-change differences in gene expression for the 38 transcripts in the extracellular matrix related canonical pathway and the 7 transcripts in the DNA binding canonical pathway identified using GSEA (Table S12), log2 fold change differences between parent and flow-sorted MDA MB 231 cells (green bars) and between parent and pore-selected MDA MB 231 cells (red bars) were plotted

To determine whether there were any specific functions associated with the 547 small cell gene set (**Table S10**), they were compared with the 1329 GSEA [30] defined ‘Canonical Pathway’ gene sets. This analysis led to the identification of two functional categories (Figure 5(a); Table S12). There were 38 transcripts associated with the ‘Matrisome’, which is a collection of extracellular matrix proteins and associated factors identified by Naba et al.(Figure 5(a))[31] In addition, 7 transcripts coded for DNA-binding proteins that were previously associated with meiosis in the Reactome Pathway Knowledgebase (Figure 5(a)) [32]. Within the extracellular matrix associated transcripts, 29 were expressed at lower levels in both Sel and Flow-Sorted cells, and 9 were more highly expressed (Figure 5(b)). The changes in extracellular matrix protein expression might contribute to the decreased adhesiveness of both Sel and Flow-Sorted cells relative to Parent cells (Figure 1(f)), independent of the significant decrease in focal adhesion density observed for the Sel populations [26]. Amongst the DNA binding proteins, the nucleosome histone H2A type 1-C, histone H2B type 1-D, histone H2B type 1-K, histone H2B type 2-E and histone H4, were all expressed at lower levels in Sel and Flow-Sorted cells relative to Parent (Figure 5(b)). Histone H3 was also lower in the Sel and Flow-Sorted populations relative to Parent cells, but did not reach the minimum 1.5-fold change cut-off in the Sel vs Parent comparison. The lower levels of nucleosome histone expression could alter patterns of gene expression that contribute to the relatively small size of Sel and Flow-Sorted cells. Alternatively, since both pore-selection and flow sorting led to the isolation of cells with smaller nuclei that contained fewer chromosomes, the reduced histone expression may simply reflect their lower DNA content relative to Parent cells [26].

### Pore-selected cells give rise to rapidly growing orthotopic xenograft tumours

**Figure 6.**
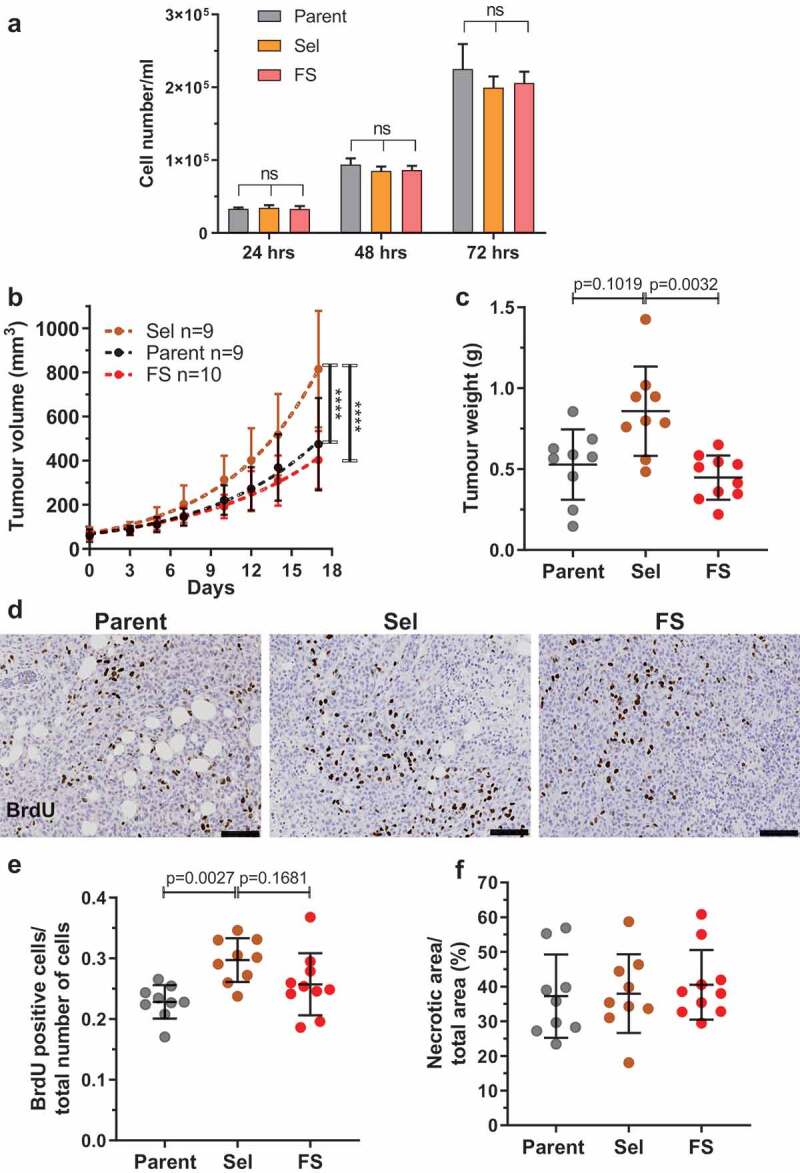
Pore-selected MDA MB 231 breast cancer cells grow faster as orthotopic xenograft tumours. (a). Proliferation of Parent, Sel and FS MDA 231 cells was determined by plating 30,000 cells per well of 12-well plate and counting cell numbers after 24, 48 and 72 hours. The number of experimental replicates were n = 12 for each population at each time point. Kruskal-Wallis test with Dunn’s multiple comparisons. ns = not significant. Means ± SEM. (b). Parent, Sel and FS cells were injected at 106 cells in 50 µl PBS:matrigel into the fourth abdominal fat pad of female immuncompromised CD1-nu/nu mice. Tumour measurements from when they became palpable (day 0). Tumour volumes were quantified from calliper measurements using the formula 1/2(Length × Width2). Statistical analysis was by two-way ANOVA and *post-hoc* Tukey’s multiple comparison test. (**** = p < 0.0001). (c). Mean tumour weights (± SD) from each mouse after 17 days. Kruskal-Wallis test with Dunn’s multiple comparisons showing adjusted p values. (d). Representative images of anti-BrdU-stained tumour sections for Parent, Sel2 and FS1 MDA MB 231 tumours from mice that had been injected intraperitoneally with BrdU two hours prior to sacrifice. Scale bar = 100 µm. (e). Mean proportion of BrdU positive cells per total cells counted (± SD) in Parent, Sel2 and FS1 MDA MB 231 tumours. Kruskal-Wallis test with Dunn’s multiple comparisons showing adjusted p values. (f). Mean percentage necrotic area (± SD) determined visually in haematoxylin and eosin stained sections of Parent, Sel2 and FS1 MDA MB 231 tumours. Kruskal-Wallis test revealed no significant differences

To determine whether there were differences in proliferation between the Parent, Sel and Flow-Sorted MDA MB 231 cells, 30,000 cells per well were plated into 12-well plates, and the number of cells per well counted after 24, 48 and 72 hours (Figure 6(a)). This analysis revealed no significant differences in the *in vitro* proliferation between any of the MDA MB 231 populations.

The tumorigenic properties of Parent, Sel2 and FS1 MDA MB 231 populations were examined by injecting 106 cells suspended in 50% Matrigel into the fourth abdominal mammary fat pads of female CD1-nude mice, and then measuring tumour dimensions after they became palpable (day 0) over 17 subsequent days. Using the formula of tumour volume = ½(length X width^2^) [33] to compare tumour growth, the volumes of Sel2 tumours were significantly greater than those grown from injected Parent or FS1 cells (Figure 6(b)). Each tumour was removed and weighed at the experimental endpoint, revealing that Sel2 tumours were significantly larger than FS1 tumours, and trended towards being larger than tumours grown from Parent cells (Figure 6(c)). To characterize *in vivo* tumour cell proliferation, each mouse was injected intraperitoneally with BrdU 2 hours prior to sacrifice, and formalin-fixed paraffin-embedded tumour sections were stained with anti-BrdU antibody to assess DNA synthesis (Figure 6(d)). For each mouse, the ratio of mean BrdU positive cells per total cells per field has been plotted, revealing significantly higher proliferation in Sel2 tumours compared to Parent tumours, and a trend towards greater proliferation compared to FS1 tumours (Figure 6(e)). In all three populations, there were comparably large necrotic regions (Figure 6(f)). Taken together, these results indicate that the properties associated with selection through narrow diameter pores promote *in vivo* tumour growth.

### Mathematical model of confined migration reveals interaction of reduced size and stiffness

**Figure 7.**
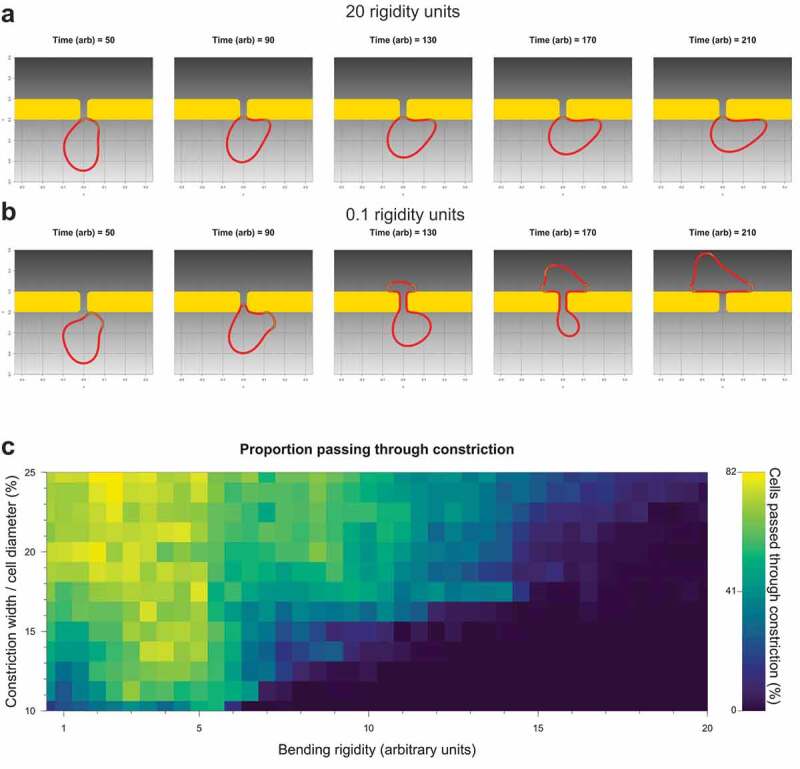
Mathematical modelling of migration through constrictions reveals interplay of cell size and elasticity. (a). Simulation time series of a cell with 16.25% constriction width/cell diameter ratio and 20.0 rigidity units failing to pass through a fixed gap. (b). Simulation time series of a cell with 16.25% constriction width/cell diameter ratio and 0.1 rigidity units passing through a fixed gap. (c). Heatmap of the percentage of cells that successfully passed through the fixed gap at constriction width/cell diameter ratios ranging from 10 to 25% at 1% intervals, and bending rigidity units ranging from 0 to 20 at 0.5 rigidity units, in 100 independent simulations per size-rigidity pairing

Since Flow-Sorted MDA MB 231 cells had 3D volumes and 2D areas equivalent to Pore-Selected cells, but were no better at moving through 3 µm pores than Parent cells [26], a question is whether the smaller size of Pore-Selected cells contributes to their ability to move through narrow gaps, or if their reduced stiffness would be sufficient. To answer this question, a previously described mathematical model of chemotactic cell migration through constrictions [34,35] was adapted to include a Helfrich bending energy (as described in Eliot *et al* [36].) as a proxy for cell stiffness to allow for variations of the cell boundary bending rigidity to be varied experimentally. One hundred simulations were run for each pair of 40 constriction gap width to cell diameter ratios, varying from cells being 10 times larger in diameter than the gap width (10%) to 4 times larger than the gap width (25%), versus 13 bending rigidity values ranging from 0 to 20 arbitrary units. In total, 52,000 simulations were run to examine how the ratio of constriction width to cell diameter interacted with bending rigidity *(i.e*. cell stiffness) on the proportion of cells successfully passing from low (light grey) to high chemoattractant (dark grey) through narrow gaps (Figure 7(a,b); videos S1,S2). For comparison, the ratio of the 3 µm pore gap width used for selection to the experimentally determined diameters of Parent MDA MB 231 or MDA MB 435 [26] was 17%, approximately at mid-point of the model. These simulations revealed that there is a positive interaction between the two variables, such that the greatest increase in cells succeeding at passing through narrow gaps occurs when cell size approaches the constriction width and when rigidity is decreased (Figure 7(c)). For the stiffest cells (~17 to 20 bending rigidity arbitrary units), smaller cell size does not aid passage, while for large cells (*e.g*. gap width = 10% of cell diameter), only very large decreases in bending rigidity enable passage through constrictions (Figure 7(c)). For gap width/cell diameter ratios comparable to MDA MB 231 or MDA MB 435 cells moving through 3 µm pores (~17%), decreasing bending rigidity by 33% from 18 to 12 bending rigidity units, equivalent to the decrease in stiffness of Sel MDA MB 231 isolates relative to Parent cells, increased successful cell passage through gaps from 5% to 40%. Interestingly, very soft large cells (~1 bending rigidity units and 10% gap width/cell diameter ratio) were less successful at passing through constrictions than slightly stiffer cells (~5 bending rigidity units), indicating a requirement for some cell stiffness for movement through confined environments. These results reveal that there is a ‘sweet spot’ of cell size and stiffness for passage through physical constrictions. The imposed pressure of selection for reduced cell size would likely be limited by a positive selection for proliferation competency, while the modelling revealed an additional lower limit to decreased cell stiffness that was necessary for efficient passage through narrow gaps (Figure 7(c)). By imposing the 3 µm constriction on cells undergoing migration, the mathematical modelling supports the conclusion that both Ras/Raf/MEK/ERK MAPK-independent small size and Ras/Raf/MEK/ERK MAPK-dependent decreased stiffness were co-selected to enable Sel MDA MB 231 and MDA MB 435 cells to move through confined environments.

## Discussion

With the goal of selecting soft cells from the dispersed distribution of cell elasticities within populations of MDA MB 231 and MDA MB 435 tumour cells, we used tissue culture inserts with membranes that have 3 µm diameter micropores that significantly impair cell movement [26]. Although the majority of cells cannot pass through 3 µm pores, multiple consecutive rounds of selection allowed for the isolation of populations of cells that were significantly better at moving through the narrow diameter gaps in the insert membranes. By comparing their properties with the starting Parent cells, and with three separate populations of cells isolated by flow cytometry, we were able to deduce which properties did or did not contribute to movement through small pores. The major factor that was consistently linked with movement through narrow gaps was decreased cell stiffness, which resulted from relatively higher Ras/Raf/MEK/ERK MAPK signalling and consequent changes in the actin cytoskeleton. Using selective chemical MEK inhibitors, we were able to show that the properties of Pore-Selected cells were all reversed by inhibition of MEK signalling, including actin cytoskeleton changes, reduced focal adhesion densities, increased cell migration velocity and reduced cell stiffness. Conversely, by expressing activated versions of upstream Ras/Raf/MEK/ERK MAPK regulators, we found that Parent MDA MB 231 and MDA MB 435 cells were altered to resemble Pore-Selected cells, with changed cytoskeleton organization, reduced focal adhesion density and reduced cell stiffness.

In this study, we further characterized how cells selected on the basis of movement through narrow diameter pores or by flow sorting for small size, differed from their originating Parent cell lines. Using high content image analysis to classify cells into three morphology categories, we found that pore-selection had the greatest effect on change the distributions of shape classes towards an elongated morphology and away from an irregular morphology, albeit which remained the largest shape category (Figure 1(d,e)). Flow-sorting resulted in a similar shift from irregular to elongated morphologies, although this change did not reach statistical significance. These results suggested that the change in morphology classes was influenced the combined factors of cell size and Ras/Raf/MEK/ERK MAPK signalling. Similarly, the relative adhesiveness of Pore-Selected cells was significantly lower than Parent MDA MB 231 or MDA MB 435 cells (Figure 1(f,g)). There was a trend towards reduced adhesion of Flow-Sorted FS cells that did not reach statistical significance (Figure 1(f)). Given that the focal adhesion density in Pore-Selected cells was significantly lower in Sel populations than either Parent or FS cells [26], it seems likely that the reduced adhesion of Pore-Selected cells is the result of both small cell size and lower focal adhesion density resulting from the higher Ras/Raf/MEK/ERK MAPK signalling.

In order to identify how the mechanistic basis for the differing phenotypes of the Parent, Pore-Selected and Flow-Sorted cells, we performed RNA sequencing to characterize their respective transcriptomes [27]. Plotting the number of mean sequence reads for MDA MB 231 Pore-Selected cells versus Parent (Figure 2(a)) or Pore-Selected versus Flow-Sorted (Figure 2(b)) helped to visualize the number and magnitude of the differences. Similar plotting of mean sequence reads from MDA MB 435 Pore-Selected versus Parent cells (Figure 2(d)) revealed the greater number and magnitude of the gene expression differences between these populations. Multiple comparisons of each paired data set revealed a core gene expression signature of 214 transcripts linked with the phenotypes of the Pore-Selected cells that differentiated them from Parent or Flow-Sorted cells [26]. Using GSEA [30], we determined that many of the 214 genes were also contained in gene sets associated with changes in Ras/Raf/MEK/ERK MAPK signalling (Figure 3(a)). To extend these observations, we also plotted mean sequence reads for Flow-Sorted versus Parent MDA MB 231 cells (Figure 4(a)), and then compared the list of genes that were significantly altered by at least 1.5 fold with the gene set from the Pore-Selected versus Parent comparison to reveal 547 transcripts associated with small cell size (Figure 4(b)). Although there should be no overlap between the invasive gene set (Figure 2(c)) and the small gene set (Figure 4(b)) because of the manner of the comparisons, there were actually 41 transcripts in common (Figure 4(c)). The reason for this was that there were statistically significant and sufficiently large differences in sequence reads in the Flow-Sorted versus Parent comparison that were even larger differences in the Pore-Selected versus Parent comparison (Figure 4(d)) that resulted in these genes being included in both gene sets. As a result, these genes may contribute to both regulating cell size and promoting deformability.

By comparing the 547 small cell gene set, including the 41 genes that overlapped with the 615 gene set associated with cell softening, with the 8 major gene set collections in GSEA, the most significant matches were in the Canonical Pathways collection, which represents various biological processes compiled by domain experts including BioCarta, KEGG [37] and Reactome [38]. The most significant matches were with ‘Matrisome’ type proteins identified by Naba et al [31]. and Reactome gene sets largely associated with meiosis, which were almost entirely composed of transcripts encoding for the nucleosome histones H2A, H2B and H4 (Figure 5(a,b)). Histone H3 transcripts were also expressed at lower levels in Pore-Selected and Flow-Sorted cells relative to Parent MDA MB 231, but did not reach the statistical and fold-change cut-offs to be part of the small cell gene set. The changes in matrisome gene expression may contribute to the reduced adhesion of Pore-Selected and Flow-Sorted cells relative to Parent cells (Figure 1(f,g)). The relatively lower expression of nucleosome transcripts may be a consequence of the reduced number of chromosomes in Pore-Selected and Flow-Sorted cells compared to Parent MDA MB 231 cells, suggesting that there is a mechanism regulating histone expression that is sensitive to cellular DNA content.

Under the conditions of *in vitro* cell culture, there were no differences in the growth rates of Parent, Pore-Selected or Flow-Sorted over three days (Figure 6(a)). In fact, there was even a slight trend towards reduced proliferation of the Pore-Selected and Flow-Sorted cells relative to Parent cells, albeit insufficient to achieve statistical significance. Given that there is a significant role of the Ras/Raf/MEK/ERK MAPK pathway in the regulation of cell proliferation, these results suggest that the Parent cells have achieved an optimal level of basal Ras/Raf/MEK/ERK MAPK signalling for proliferation, and that the higher Ras/Raf/MEK/ERK MAPK signalling in Pore-Selected cells that contributes to significant changes in elasticity is unable to affect proliferation. Following the injection of Parent, Pore-Selected and Flow-Sorted cells into the mammary fat pads of immunocompromised mice, tumour growth rates were significantly faster for the Pore-Selected cells based on their calculated volumes (Figure 6(b)). In addition, there was a trend towards greater tumour weights (Figure 6(c)) and greater levels of proliferation (Figure 6(d,e)). One possibility is that the increased invasive ability of the Pore-Selected cells enabled more rapid tumour growth, consistent with our previous observations in pancreatic ductal adenocarcinoma cells [39]. An alternative explanation is that the Pore-Selected cells proliferate more rapidly, relative to the Parent and Flow-Sorted cells, due to sub-optimal growth conditions *in vivo* that favour responsiveness to high Ras/Raf/MEK/ERK MAPK signalling.

Pharmacological inhibition of MEK signalling altered actin cytoskeleton structures, focal adhesion densities, cell elasticity and invasion through dense 3D collagen [26], consistent with these properties being important contributors to the ability of Pore-Selected cells to move through narrow gaps. Although small cell size was not sufficient to confer cells with this ability, the relatively small volumes of Pore-Selected cells suggests that cell size is an important factor. Since MEK inhibition for 24 hours did not increase cell diameters, in fact there was a trend towards even smaller diameters likely due an accumulation of cells arrested in the G1 cell cycle phase, we could not experimentally manipulate cell size to examine its contribution to movement through narrow diameter pores or dense collagen matrices. Instead, we employed mathematical modelling to examine the interaction of cell-to-gap ratio with cell bending rigidity in determining the efficiency of movement through constraints. The modelling indicated that there was cooperation between reduced cell size and stiffness in enabling movement through narrow diameter gaps, such that the passage of large cells was improved if they were made softer, and soft cells were more efficient if their size approached the gap width (Figure 7(c)). There were some caveats to this relationship, such as the reduced success of the largest cells actually at movement through pores if they were very soft.

Our positive-selection based approach succeeded in the objective of enriching for cells that were softer than the ‘typical’ cell from the starting Parent populations. For every property that we examined, the distribution of values around the mean in Pore-Selected cells fell within the distribution of values for Parent cells. However, in several cases including cell diameter and pMEK levels, there were left or rightward shifts in the distribution curves. These observations are consistent with the interpretation that Pore-Selection led to the isolation of pre-existing cells from the original Parent populations, rather than Pore-Selection causing changes in cells as a result of the physical stresses they encountered. Consistent with this conclusion, we found that the properties associated with Pore-Selection were stable over time, being continuously present over multiple cell passages. We propose that stable and heritable epigenetic differences in a sub-population of cells results in higher levels of constitutive Ras/Raf/MEK/ERK MAPK activity. The imposition of the selective pressure of pore migration enriched for this population of high Ras/Raf/MEK/ERK MAPK signalling cells due to the coincidence of decreased cell stiffness. We predict that if it were possible to select specifically for high Ras/Raf/MEK/ERK MAPK signalling from the Parent cell population, then cell softness would be co-selected.

## Material and methods

### Cell culture

MDA MB 231-luc-D3H2LN and MDA MB 435 cell lines were grown in HyClone MEM/EBSS media (GE Healthcare Life Sciences, 11541871), supplemented with 10% foetal bovine serum (FBS) (Gibco, 10270), 2 mM L-glutamine (Gibco, 25030–032), 10 U/ml penicillin and 10 µg/ml streptomycin (Gibco, 15140–122), 1% MEM/NEAA (Thermo Fisher Scientific, 11140,035), 1% Sodium Pyruvate (Thermo Fisher Scientific, 11360070). Cell identities were validated by the Cancer Research UK Beatson Institute Molecular Services using the GenePrint 10 system STR multiplex assay (Promega) that amplifies 9 tetranucleotide repeat loci and Amelogenin gender determining marker. All cell lines were routinely tested for mycoplasma by the Cancer Research UK Beatson Institute Molecular Services.

Independent MDA MB 231 or MDA MB 435 Pore-Selected (Sel) populations were established by seeding 1 × 10^6^ cells in 10 ml serum-free medium on 3 μm pore membranes in 7.5 cm cell culture inserts (Corning, Fisher Scientific, 3420). Inserts were placed in 10 cm dishes containing 10 ml serum-containing medium, and left for five days in standard tissue culture conditions to allow cells to migrate through the pores. The inserts were removed, media changed and plates placed back in the incubator to expand the selected cell populations. The selection process was repeated twice more as described above and in Rudzka et al [26]. to establish three independent selected populations.

Independent small diameter MDA MB 231 flow cytometry sorted (Flow-Sorted; FS) populations were obtained by gating with low forward scatter (FSC) and side scatter (SSC) parameters using a FACSAria Fusion sorter (BD Biosciences, Oxford UK). FS cells were grown using standard tissue culture conditions to expand the isolated sorted cell populations, followed by two additional rounds of sorting as described above and in Rudzka et al [26]. to establish three independent flow sorted populations.

### Morphological cell properties

To determine cell morphological features, an Operetta High-Content Imaging System was used. Prior to cell imaging, cells were seeded in black 96-well plates at 2 × 10^4^ cells per well and grown overnight. After incubation, cells were fixed with 4% para-formaldehyde (w/v) (PFA) (EMS, 15,710) in PBS for 15 minutes and permeabilized with 0.5% (v/v) Triton X-100 (Thermo Fisher Scientific, 28314) for 5 minutes at room temperature. Cells were incubated with 0.15 µg/ml DAPI (Sigma, D9542) for 20 minutes at room temperature and subsequently with 50 µl of 1:5000 dilution of Cellomics® Whole Cell Stain (WCS) (Thermo Fisher Scientific, 8303401) for 30 minutes. Plates were imaged on an Operetta High-Content Imaging System and data was analysed using a Columbus™ Image Data Storage and Analysis System (PerkinElmer) as described in Rudzka et al [26].

### Cell detachment assay

Cell were seeded at 3 × 10^5^ cells per well in 12-well plates and left overnight in standard tissue culture conditions. Cells were washed once with PBS, then 500 µl of 2 U/mg Dispase was added per well and subsequently left for 5 minutes at 37 C. 500 µl was removed and cell numbers were measured using a CASY® Cell Counter. To measure the number of cells that remained attached to the bottom of plates, 500 µl of 2 U/mg Dispase was added and left until cells detached from the plates. The percentage of attached cells was normalized to the total number of cells.

### RNA-seq

RNA Sequencing and data analysis was performed as described in Rudzka et al [26]. and Rudzka *et al* [27].

### Proliferation assay

Cells were seeded in triplicate at 3 × 10^3^ cells per well in 12-well plates. Cell numbers were measured at 24, 48 and 72 hours. Cells were trypsinized and counted using a CASY® Cell Counter.

### Orthotopic injection of breast cancer cells into mammary fat pads of nude mice

MDA MB 231 breast cancer cells were injected into the mammary fat pads of athymic nude female CD1 mice obtained from Charles River. Seven week old mice were injected with 1 × 10^6^ cells in 50 µl of a 1:1 Matrigel:PBS solution into the fourth abdominal fat pad. Tumour growth was monitored over the course of study and animals were sacrificed when tumour size reached 15 mm. Mice were injected intraperitoneally with BrdU two hours prior to sacrifice. Primary tumour and organs were harvested, paraffin embedded and stained with haematoxylin and eosin (H&E) or antibodies against BrdU. Histology sample processing was performed by the Beatson Histology Service. Slides were analysed using Leica Biosystems software. All mouse experiments were carried out under ethical review (University of Glasgow) and UK Home Office licence permission in dedicated facilities proactive in environmental enrichment.

### Modelling cell movement through constrictions

A previously described computational model, which simulates pseudopod-centred cell migration and chemotaxis in the presence of obstacles [34,35], was reformulated to include a Helfrich bending energy as described in [36], thereby allowing control of the bending rigidity of the cell boundary.

## Supplementary Material

Supplemental Material

Supplemental Material
