# Supplementary material for: Selection of established tumour cells through narrow diameter micropores enriches for elevated Ras/Raf/MEK/ERK MAPK signalling and enhanced tumour growth
Source: Small GTPases. 2020 Jun 22;12(4):294–310. doi: 10.1080/21541248.2020.1780108 (PMC8204978; doi:10.1080/21541248.2020.1780108)
Supplement: Supplemental Material [file KSGT_A_1780108_SM3666.zip › Supplemental Movie_Table Legends.docx]

**Selection of established tumour cells through narrow diameter micropores enriches for elevated Ras/MAPK signalling and enhanced tumour growth.**

**Supplemental Movie and Table Legends.**

**Movie S1 (related to Fig 7A).** Simulation of a cell with 16.25% constriction width/cell diameter ratio and 20.0 rigidity units passing through a fixed gap in response to a chemoattractant gradient from low (light grey) to high (dark grey).

**Movie S2 (related to Fig 7B).** Simulation of a cell with 16.25% constriction width/cell diameter ratio and 0.1 rigidity units passing through a fixed gap in response to a chemoattractant gradient from low (light grey) to high (dark grey).

**Table S1**. **RNA sequence differences between Parent and Selected MDA MB 231 populations.** RNA sequence reads comparing Parent MDA MB 231 with Sel2 and Sel3 MDA MB 231 populations. Cut-offs used were fold-change (FC) > 1.5 times and p < 0.05.

**Table S2**. **RNA sequence differences between Selected and Flow Sorted MDA MB 231 populations.** RNA sequence reads comparing Sel2 and Sel3 MDA MB 231 with FS1 and FS2 MDA MB 231 populations. Cut-offs used were fold-change (FC) > 1.5 times and p < 0.05.

**Table S3**. **RNA sequence differences associated with constrained migration ability in MDA MB 231 cells.** RNA sequence reads comparing Parent MDA MB 231 with Sel2 and Sel3 MDA MB 231 from Supplemental Table S1, with the Sel2 and Sel3 MDA MB 231 comparison with FS1 and FS2 MDA MB 231 populations from Table S2. Cut-offs used were fold-change (FC) > 1.5 times and p < 0.05.

**Table S4**. **RNA sequence differences between Parent and Selected MDA MB 435 populations.** RNA sequence reads comparing Parent MDA MB 231 with Sel1 and Sel2 MDA MB 435 populations. Cut-offs used were fold-change (FC) > 1.5 times and p < 0.05.

**Table S5. Pore invasion gene set.** RNA sequence reads comparing the constrained migration gene set for MDA MB 231 cells from Table S3 with the MDA MB 435 comparisons between Parent and Selected populations in Supplemental Table S4.

**Table S6. Gene set enrichment analysis oncogenic signature gene sets for MDA MB 231 constrained migration RNA sequencing results.** The top 10 oncogenic signature gene sets identified from the RNA sequencing comparisons in Table S3.

**Table S7. Gene set enrichment analysis oncogenic signature gene sets for the consensus invasion gene set from MDA MB 231 and MDA MB 435 comparisons.** The top 10 oncogenic signature gene sets identified from the RNA sequencing comparisons in Table S4.

**Table S8. KRAS, BRAF, and/or Mitogen Activated Protein Kinase (MAPK)-linked oncogenic signature genes.** RNA sequencing reads for the subset of the pore invasion gene set identified in Table S5 that were associated with KRAS, BRAF and/or MAPK signalling in gene set enrichment analysis oncogenic signatures.

**Table S9**. **RNA sequence differences between Parent and Flow Sorted MDA MB 231 populations.** RNA sequence reads comparing Parent MDA MB 231 with FS1 and FS2 MDA MB 231 populations. Cut-offs used were fold-change (FC) > 1.5 times and p < 0.05.

**Table S10**. **RNA sequence differences associated with small cell size of MDA MB 231 cells.** RNA sequence reads comparing Parent MDA MB 231 with Sel2 and Sel3 MDA MB 231 from Table S1, with the Parent MDA MB 231 comparison with FS1 and FS2 MDA MB 231 populations from Table S9. Cut-offs used were fold-change (FC) > 1.5 times and p < 0.05.

**Table S11**. **RNA sequence differences common between constrained migration and associated with small cell size of MDA MB 231 cells.** RNA sequence reads associated with constrained migration in Table S3 were compared with the RNA sequence reads associated with small cell size in Table S10.

**Table S12. Gene set enrichment analysis canonical pathway gene sets for the small cell size gene set from MDA MB 231 comparisons.** The top 10 oncogenic signature gene sets identified from the RNA sequencing comparisons in Table S10.
